# Pantaloon Hernia: Obstructed Indirect Component and Direct Component with Cryptorchidism

**DOI:** 10.1155/2016/1461425

**Published:** 2016-08-04

**Authors:** Mohan Kumar Kariappa, Vivek Harihar, Ashwini Rajareddy Kothudum, Vivekanand Kedarlingayya Hiremath

**Affiliations:** Department of Surgery, Sri Devaraj Urs Medical College and Research Centre, Kolar 563101, India

## Abstract

Cryptorchidism is a condition in which one or both testes have not passed down into the scrotal sac. It is categorized as true undescended testis in which testes are present in the normal path of descent, and as ectopic testis, in which testes are present at abnormal site. Common complications of cryptorchidism are testicular torsion, subfertility, inguinal hernia, and testicular cancer. Here we present a rare case of pantaloon hernia of obstructed indirect component and direct component with cryptorchidism.

## 1. Case Report

A 40-year-old male patient presented to the emergency department of RLJ Hospital with history of pain and swelling in the right inguinoscrotal region following lifting heavy metal pipe since 1 day.

## 2. O/E

The following was noticed:

(i) Abdomen was soft, and diffuse tenderness in the lower abdomen was present.
(ii) There was irreducible swelling measuring 6 × 4 cm, which was present in right inguinoscrotal region and was firm in consistency. Skin is erythematous, stretched, and shiny.

**Figure 1:**
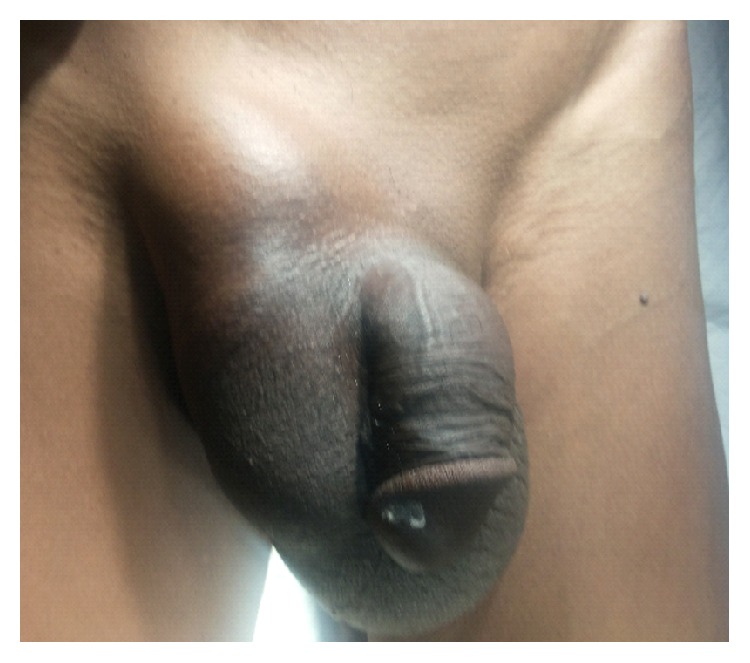
Preoperative picture.

Regarding the external genitalia,

- right testis was not palpable,
- left testis is palpable and penis is normal (Figure 1),
- in the rest of the examination, nothing was significant.

A diagnosis of right irreducible inguinal hernia with undescended testis was made.

## 3. Preoperative Findings

**Figure 2:**
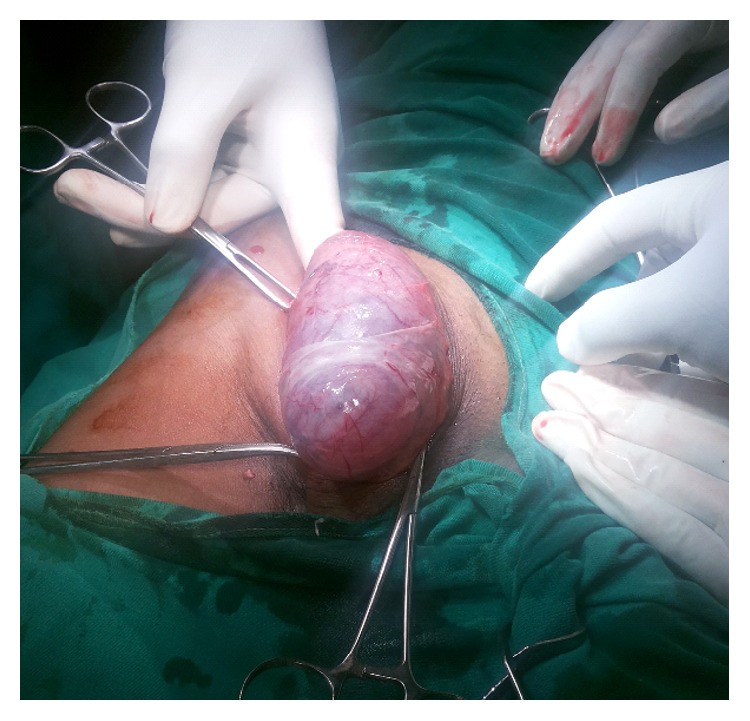
Hernia with sac.

**Figure 3:**
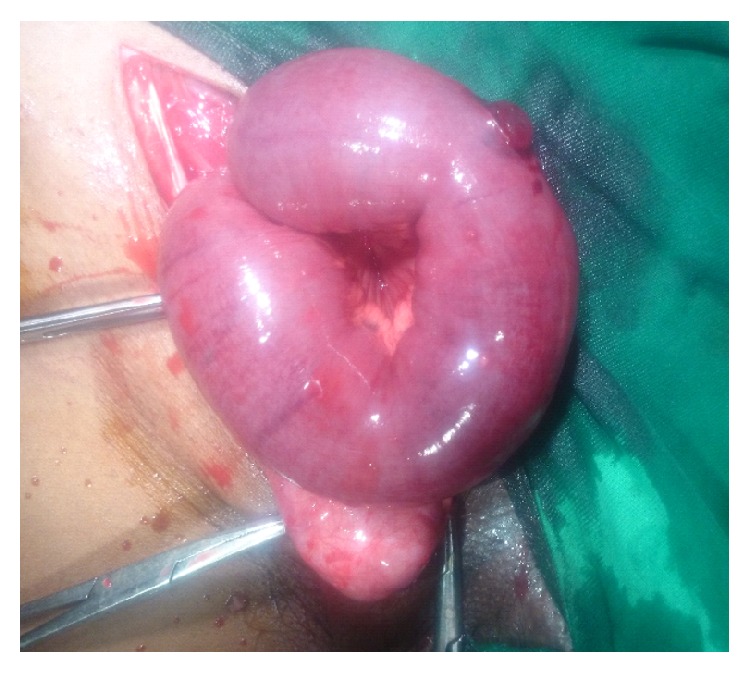
Pregangrenous bowel with atrophic testis as contents of indirect sac.

Surgical exploration revealed an indirect hernia of incomplete type (Figure 2) with pregangrenous bowel loop and atrophic testis (Figure 3). After releasing the neck, bowel loop was viable; therefore reduced and sac ligation followed by excision was done.

**Figure 4:**
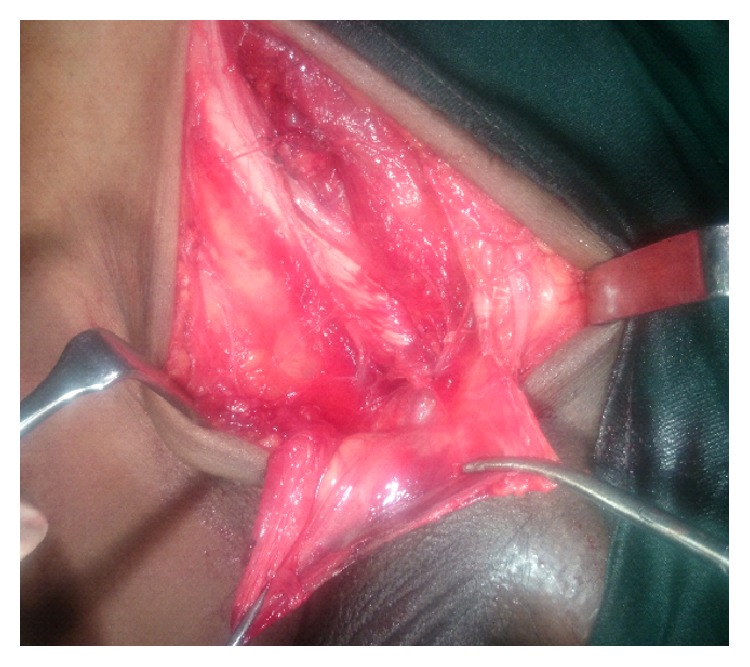
After sac ligation and excision.

**Figure 5:**
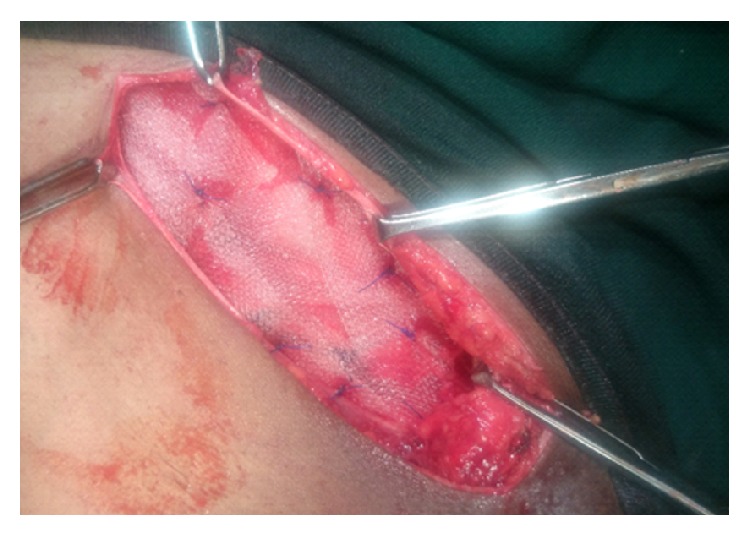
Hernioplasty.

Another direct type sac was noted with omentum as content, which was reduced (Figure 4). In view of age and atrophic testis, high orchidectomy with hernioplasty was done (Figure 5).

Patient tolerated the procedure well and the postoperative period was unremarkable.

## 4. Histopathology

**Figure 6:**
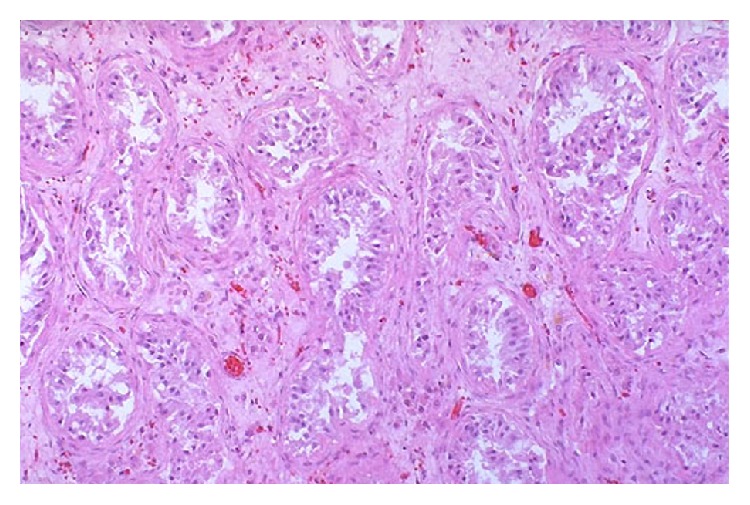
Histopathological study of atrophied testis.

Histopathology report confirmed the presence of Leydig cells, seminiferous tubule, and Sertoli cells without Testicular Germ Cell Tumors (TGCT) (Figure 6).

## 5. Discussion

An undescended testis, sometimes called a cryptorchid testis, can be found in 3% of the term newborns and in 0.5–1.0% of adults [1]. Cryptorchidism is more commonly seen in premature males and associated to genetic disorders in 10% of the cases. The causes of cryptorchidism are prematurity, spina bifida, hormonal disorders, testicular absence, or retractile testes. Smoking more than 10 cigarettes a day during pregnancy increased the risk of cryptorchidism [2]. It is concluded that the second inguinoscrotal stage of testicular descent is clearly androgen-dependent [3]. The diagnosis of cryptorchidism is made by physical examination. The diagnosis of cryptorchidism should be considered when nonpalpable testis and inguinal hernia are present. However, each patient may experience symptoms differently. Nonetheless, for inguinal hernia, the clinical presentation varies, depending on the contents of the hernial sac and the degree of herniation. Because of its varied presentation, clinical examination is often inconclusive [4]. The correct diagnosis of inguinal hernia is usually made during an inguinal hernia repair, although ultrasonography and computerized tomography have been used to identify an inguinal hernia [5].

The complications of unrepaired cryptorchidism are mainly testicular torsion, infertility, inguinal hernia, and testicular cancer. The testicles begin to lose the process of spermatogenesis if they are not in the scrotum because the scrotum is a “cooler location.” This process explains the link between cryptorchidism and infertility. In our case, inspite of old age, histopathology report confirmed the absence of tumor. Because the incidence of testicular cancer generally increases in cryptorchid testes, careful follow-ups are essential [6, 7].

Usually, cryptorchidism resolves without any intervention before the age of 6 months. Surgical repair for cryptorchidism will be carried out if the testicles have not descended. Studies have shown that individuals who had corrective surgery after the age of 13 years had an incidence rate of 5.4%, whereas those who were treated before 13 years had an incidence rate of 2.23% [8]. Histopathology report confirmed the presence of Leydig cells, seminiferous tubule, and Sertoli cells without TGCT. The treatment of cryptorchidism reduces the risks of infertility and gonadal neoplasia. Surgical repair for cryptorchidism will result in earlier detection of an eventual tumor.

It is difficult to understand the surgical anatomy of inguinal hernias, but once the surgical exploration is performed, surgical repair is simple. It is controversial whether a contralateral orchidopexy is needed or not. Furthermore, the incidence of testicular cancer does not decrease with fixed testes [9, 10]. In our case, the contralateral orchidopexy was not performed.

## 6. Conclusion

This case is particularly notable because of the unusual presentation of cryptorchid testis as an obstructed inguinal hernia at the age of 40 years. The patient remained asymptomatic for 40 years. To our knowledge, the present case represents one of the very few cases of cryptorchid testis revealed at the age of 40 years with an inguinal hernia.

*“The surgeon must always be alert to the possibility of cryptorchid testis during a surgical exploration of an inguinal hernia.”*
